# Evaluation of Preferred Language and Timing of COVID-19 Vaccine Uptake and Disease Outcomes

**DOI:** 10.1001/jamanetworkopen.2023.7877

**Published:** 2023-04-12

**Authors:** Nasreen S. Quadri, Greg Knowlton, Gabriela Vazquez Benitez, Kirsten R. Ehresmann, Amy B. LaFrance, Terese A. DeFor, M. Kumi Smith, Erin M. Mann, Jonathan D. Alpern, William M. Stauffer

**Affiliations:** 1 Department of Medicine, University of Minnesota, Minneapolis; 2 Departments of Medicine and Pediatrics, Infectious Diseases and International Medicine, University of Minnesota, Minneapolis; 3 Center for Global Health and Social Responsibility, University of Minnesota, Minneapolis; 4 HealthPartners Institute, Bloomington, Minnesota; 5 Division of Epidemiology and Community Health, School of Public Health, University of Minnesota, Minneapolis

## Abstract

**Importance:**

Beyond traditional race and ethnicity demographic characteristics, additional discrete data variables are needed for informed health interventions in the US.

**Objective:**

To examine whether COVID-19 vaccine uptake patterns and associated disease outcomes differ among language preference groups.

**Design, Setting, and Participants:**

A cohort study of 851 410 individuals aged 18 years or older in a large multispecialty health system in Minnesota and western Wisconsin was conducted between December 15, 2020, and March 31, 2022.

**Exposure:**

Self-identified language preference and limited English proficiency (LEP) as measured by interpreter need were used to create subgroups using US census categories and attention to capture languages known to represent refugee groups.

**Main Outcomes and Measures:**

The primary outcome was COVID-19 vaccination uptake rates and time to first vaccine. Secondary outcomes were rates of COVID-19–associated hospitalization and death.

**Results:**

Most of the 851 410 participants (women, 493 910 [58.0%]; median age, 29 [IQR, 35-64] years) were US-born English speakers; 7.5% were born in other countries, 4.0% had a language preference other than English (LPOE), and 3.0% indicated LEP as measured by interpreter need. Marked temporal clusters were observed for COVID-19 vaccination uptake, hospitalizations, and deaths associated with primary series vaccine eligibility, booster availability, and COVID-19 variants. Delayed first-dose vaccine was observed with LPOE (hazard ratio [HR], 0.83; 95% CI, 0.82-0.84) and interpreter need (HR, 0.81; 95% CI, 0.80-0.82) compared with those with English language preference and proficiency. Patients with LPOE were approximately twice as likely to be hospitalized (rate ratio [RR], 1.85; 95% CI, 1.63-2.08) or die (RR, 2.13; 95% CI, 1.65-2.69). Patients with LEP experienced even higher rates of hospitalization (RR, 1.98; 95% CI, 1.73-2.25) and COVID-19–associated death (RR, 2.32; 95% CI, 1.79-2.95). Outcomes varied for individual language preference groups.

**Conclusions and Relevance:**

In this study, delayed time to first-dose vaccine was associated with increased COVID-19 hospitalization and death rates for specific LPOE and LEP groups. The findings suggest that data collection of language preference and interpreter need provides actionable health intervention information. Standardized system-level data collection, including at a national level, may improve efficient identification of social groups with disproportionate health disparities and provide key information on improving health equity in the US.

## Key Points

**Question:**

Are there linguistic disparities in COVID-19 vaccine uptake and disease outcomes based on self-reported preferred language and interpreter need?

**Findings:**

In this cohort study of 851 410 individuals between December 2020 and March 2022, self-identified language preference other than English and limited English proficiency, as measured by interpreter need, were both associated with delayed time to first vaccine dose and increased rates of COVID-19–associated hospitalization and death among specific language preference groups. Marked temporal clusters were observed for COVID-19 vaccination uptake, hospitalizations, and deaths associated with primary series vaccine eligibility, booster availability, and COVID-19 variants.

**Meaning:**

The findings of this study suggest that disaggregated data collection of preferred language and interpreter need is essential to identify and address barriers to care to improve health disparities in the US.

## Teaser

This cohort study examines the differences in timing of COVID-19 vaccine administration among individuals with language preference other than English.

## Introduction

Language justice is a framework encompassing the fundamental right of individuals to communicate in their preferred language in an increasingly multilingual society. Health care communication to the public has been challenging during the COVID-19 pandemic.^1,2^ Gaps in effective messaging deepen along linguistic lines. One study found that people with a language preference other than English (LPOE) are at higher risk of testing positive for COVID-19 regardless of age, race and ethnicity, or other social factors.^3^ Unmet language access needs are a key barrier to high-quality health care and health equity across racial and ethnic groups.^4^ Linguistic minority groups are vulnerable to epistemic injustice—the communicative contextual disadvantage within which power asymmetries and language discordance affects the exchange of health information.^5^ Patients with limited English proficiency (LEP) are less likely to have a clinical home or to have received preventive care in the prior year, even after adjusting for sociodemographic factors.^6^ In the US context, LPOE and LEP are associated with worse health outcomes.^7^

According to the 2019 American Community Survey, among 65 million people in the US who speak a language other than English at home, 39% report limited English proficiency (ie, speak English less than very well).^8^ Language groups vary by local context and migration patterns, forced or voluntary. Notably, one-third of individuals immigrating to the US when aged 12 years or older reported not being fluent in English, although rates varied based on educational attainment, race and ethnicity, and age at immigration.^9^ Health care systems and public health organizations are adapting to a changing linguistic landscape. Medically trained interpreters improve patient health care outcomes.^10^ Offering such services is a legal requirement (Section 1557 of the Affordable Care Act).^11,12^ Nonetheless, professional medical interpreters are underused, leading to differences in diagnostic care, communication errors, and decreased patient satisfaction.^10^ Providing access to health information through culturally and linguistically appropriate methods surpasses technical translation to include how health care systems and public health agencies accommodate unique cultural contexts.^5^

Scant literature exists on linguistic disparities in COVID-19 vaccine uptake. Vaccine coverage disparities are well documented in the Centers for Disease Control and Prevention social vulnerability index, including socioeconomic status, household composition and disability, racial and ethnic minority status and language, and housing and transportation.^13^ Language barriers may prevent people from learning vaccine risks and benefits, eligibility, and vaccination locations.^14^ Furthermore, minoritized groups may have experienced poor health care and discrimination due to cultural insensitivity, creating mistrust and reducing health care–seeking behavior.^15^ Patients with LEP vary in their trust, health communication preferences, and vaccination access and acceptance, leading to cultural and linguistic barriers.^16,17,18^ A self-reported national survey found heterogeneity in vaccination intent, hesitancy, and coverage by nativity, race and ethnicity, and language.^19^ Studying linguistic disparities advances actionable solutions.

This report analyzes COVID-19 vaccine uptake rates and associated hospitalizations and deaths from December 15, 2020, to March 31, 2022, in one large health care system in Minnesota and Wisconsin, which serves a uniquely multilingual population. We report rates of COVID-19 vaccination inclusive of the primary series and booster doses over that period and compare time from vaccine rollout to first COVID-19 vaccine dose by language group and LEP, adjusted by age. We further explore how disaggregating race and ethnicity classifications by patient self-identified language preference groups may add valuable health information to reduce health disparities.

## Methods

The HealthPartners Research Subjects Protection Program deemed this study a public health activity not requiring human participant oversight. This study followed the Strengthening the Reporting of Observational Studies in Epidemiology (STROBE) reporting guideline for cohort studies. HealthPartners includes a large multispecialty health system serving more than 1.2 million patients in Minnesota and western Wisconsin. Data comprise patients who sought care at a HealthPartners facility between January 1, 2019, and March 31, 2022. Inclusion criteria required a patient to have sought care at least once in 2019 or 2020 and once between December 15, 2020, and March 31, 2022, and be aged 18 years or older on December 15, 2020. The study period was between December 15, 2020, and March 31, 2022. Vaccination records were obtained from electronic health records and supplemented by records from the Minnesota Immunization Information Connection to capture vaccinations outside the system. Hospitalizations for COVID-19 disease were identified based on *International Statistical Classification of Diseases and Related Health Problems, Tenth Revision* diagnosis code U07.1. Death records from the Minnesota Department of Health Office of Vital Records were used to confirm COVID-19–associated deaths statewide through March 31, 2022.

Self-identified patient demographic characteristics collected by staff at patient intake include race and ethnicity, preferred language, country of birth, and a recorded need for an interpreter (as a surrogate referred to as LEP). Patient race and ethnicity options are prepopulated, including the following categories: American Indian or Alaskan Native, Asian, Black or African American (hereafter, Black), Hispanic or Latino (hereafter, Hispanic), Native Hawaiian or Pacific Islander, White, and other. Additionally, ethnicity options are dichotomous between Hispanic or not Hispanic . Patients could select more than 1 race; those patients were categorized into 1 cohort as multiracial. The other category was a prepopulated option for the registration questions on race and ethnicity and preferred language. Language was captured by asking the patient which language they prefer to use when communicating with a health care professional. These data were supplemented by administrative records for patients/members with HealthPartners insurance. Patients who indicated either Hispanic race or Hispanic ethnicity of any race herein were classified under Hispanic; all other groupings included patients who self-identified as non-Hispanic. Based on internal analysis, nonresponders with missing data on language preference (0.7%) were categorized as English speakers, country of birth (29.7%) as born in the US, and interpreter need (1.3%) as not needing an interpreter.

The US Census Bureau and the American Community Survey collapse languages into categories based on the 1977 Classification and Index of the World's Languages and the 2016 Ethnologue: Languages of the World. Aggregation or disaggregation of languages is based on population size.^20^ Similarly, language aggregation in this study was informed by US census categories and internal discussion based on knowledge of regional population groups. Special attention was given to disaggregate language groups known to represent refugee groups, given increasing awareness of forced migration as an independent social determinant of health. HealthPartners’ catchment area has a high percentage of known refugee populations.^20,21^ Language groups with 500 or more participants were included (eMethods in Supplement 1).

### Statistical Analysis

All analyses were conducted using R, version 4.1.2 (R Foundation for Statistical Computing). Monthly age-adjusted rate ratios (RRs) and 95% CIs describe COVID-19 vaccination uptake, hospitalizations, and deaths across LPOE and LEP. These data were derived from Poisson regression models including each factor of interest by month and age groups as covariates. Overall age-adjusted RRs and 95% CIs were estimated for vaccination, hospitalization, and death to reflect proportional differences among groups. The RR estimates assume all vaccine types are equivalent. Time to first vaccine dose was analyzed using hazard ratios (HRs) and 95% CIs to capture the time in days when the first dose occurred, from December 15, 2020, through March 31, 2022. The age-adjusted time to first vaccine dose HR analysis was derived from a Cox proportional hazards model. Observations were censored at death or, if unvaccinated, at the study period end. The reference categories were English for language, White for race and ethnicity, US for country of birth, and interpreter not needed, based on the majority of the sample for each category. Comparisons were performed for race and ethnicity, LPOE, nativity status other than US, LEP, and selected language groups. Sensitivity analysis was conducted to address the large imbalance between sample size in the reference category and other categories by undersampling the reference group to equal the sample size of all nonreference categories 5000 times for each model. For this descriptive analysis, HR and RR associations were presented with 95% CIs; thresholds for statistical significance were not established.

## Results

This study included 851 410 adults, including 493 910 women (58.0%) and 357 500 men (42.0%); median age was 29 (IQR, 35-64) years. Most were US-born English speakers; 7.5% were born in other countries, 4% had LPOE, and 3% had LEP measured by interpreter need (Table 1). The population included 0.4% American Indian or Native American, 5.1% Asian, 9.0% Black, 4.1% Hispanic, 0.1% Native Hawaiian/Pacific Islander, 77.5% White, 1.5% other, and 1.2% multiracial individuals. Only 1.2% of the individuals lacked a race or ethnicity category. LEP measured by interpreter need was more common in people who self-identified as Asian (20.3%), Black (8.7%), and Hispanic (21.0%) (eTable in Supplement 1). The most common preferred languages other than English were Spanish (27.0%), Somali (19.3%), and Vietnamese (10.3%). Characteristics of individuals with LPOE, LEP, and born in countries other than the US are described in the eTable in Supplement 1. Preferred language and interpreter need data were comprehensive; data were missing for only 0.7% of the sample for preferred language and 1.3% for interpreter need. Nonresponders for language preference and interpreter need were assigned English and interpreter not needed based on inference after internal analysis. However, 30% of the study population lacked country of birth data; these individuals were assigned to US-born after additional internal analysis of demographic factors, such as race and ethnicity classification and language preference. By the end of the observation period, 19.3% of the individuals never received a dose of the COVID-19 vaccine. Our analysis revealed most patients (94%) who received messenger RNA vaccines completed the primary series within 42 days.

**Table 1.** Baseline Characteristics of Language Preference Other Than English (LPOE), Race and Ethnicity, Sex, Age by Preferred Language (Grouped into English vs non-English), Interpreter Need and Country of Birth (US-born vs Born Outside the US)

Variable	Overall (N=851 410)	Language preference		Interpreter need		Country of birth	
		English (n=817 440)	Non-English (n=33 970)	No (n=825 483)	Yes (n=25 927)	US (n=787 785)	Other (n=63 625)
Birthplace other than US, No. (%)	63 625 (7.5)	44 381 (5.4)	19 244 (56.6)	47 707 (5.8)	15 918 (61.4)	NA	63 625 (100.0)
Language preference other than English (%)	33 970 (4.0)	NA	33 970 (100.0)	8052 (1.0)	25 918 (100.0)	14 726 (1.9)	19 244 (30.2)
Interpreter need, No. (%)	25 927 (3.0)	9 (0.0)	25 918 (76.3)	NA	25 927 (100.0)	10 009 (1.3)	15 918 (25.0)
Preferred language (%)							
English	817 440 (96.0)	817 440 (100.0)	NA	817 431 (99.0)	9 (0)	773 059 (98.1)	44 381 (69.8)
Arabic	593 (0.1)	NA	593 (1.7)	204 (0)	389 (1.5)	231 (0)	362 (0.6)
Cambodian/Khmer	1266 (0.1)	NA	1266 (3.7)	190 (0)	1076 (4.2)	579 (0.1)	687 (1.1)
Chinese languages	1329 (0.2)	NA	1329 (3.9)	235 (0)	1094 (4.2)	578 (0.1)	751 (1.2)
Eastern European languages	1146 (0.1)	NA	1146 (3.4)	309 (0)	837 (3.2)	510 (0.1)	636 (1.0)
Ethiopian languages	2460 (0.3)	NA	2460 (7.2)	460 (0.1)	2000 (7.7)	503 (0.1)	1957 (3.1)
Hmong	1746 (0.2)	NA	1746 (5.1)	373 (0)	1373 (5.3)	711 (0.1)	1035 (1.6)
Myanmar (formerly Burma) languages	909 (0.1)	NA	909 (2.7)	53 (0)	856 (3.3)	98 (0)	811 (1.3)
South Asian Languages	579 (0.1)	NA	579 (1.7)	348 (0)	231 (0.9)	341 (0)	238 (0.4)
Laotian	618 (0.1)	NA	618 (1.8)	150 (0)	468 (1.8)	375 (0)	243 (0.4)
Nepali	584 (0.1)	NA	584 (1.7)	86 (0)	498 (1.9)	89 (0)	495 (0.8)
American Sign Language	712 (0.1)	NA	712 (2.1)	24 (0)	688 (2.7)	662 (0.1)	50 (0.1)
Somali	6562 (0.8)	NA	6562 (19.3)	2016 (0.2)	4546 (17.5)	2685 (0.3)	3877 (6.1)
Spanish	9166 (1.1)	NA	9166 (27.0)	1864 (0.2)	7302 (28.2)	4630 (0.6)	4536 (7.1)
Vietnamese	3511 (0.4)	NA	3511 (10.3)	422 (0.1)	3089 (11.9)	1412 (0.2)	2099 (3.3)
Other (n <500)	2789 (0.3)	NA	2789 (8.2)	1318 (0.2)	1471 (5.7)	1322 (0.2)	1467 (2.3)
Sex, No. (%)							
Female	493 910 (58.0)	471 882 (57.7)	22 028 (64.8)	476 675 (57.7)	17 235 (66.5)	455 395 (57.8)	38 515 (60.5)
Male	357 500 (42.0)	345 558 (42.3)	11 942 (35.2)	348 808 (42.3)	8692 (33.5)	332 390 (42.2)	25 110 (39.5)
Age, mean (SD), y	49.66 (18.3)	49.64 (18.4)	50.09 (17.4)	49.58 (18.4)	52.31 (17.1)	49.81 (18.5)	47.81 (16.4)
Age group (%), y							
18-30	153 375 (18.0)	148 836 (18.2)	4539 (13.4)	150 818 (18.3)	2557 (9.9)	144 333 (18.3)	9042 (14.2)
31-50	285 901 (33.6)	272 281 (33.3)	13 620 (40.1)	275 998 (33.4)	9903 (38.2)	256 740 (32.6)	29 161 (45.8)
51-64	226 217 (26.6)	217 510 (26.6)	8707 (25.6)	218 978 (26.5)	7239 (27.9)	210 816 (26.8)	15 401 (24.2)
65-84	166 369 (19.5)	160 041 (19.6)	6328 (18.6)	160 828 (19.5)	5541 (21.4)	157 477 (20.0)	8892 (14.0)
≥85	19 548 (2.3)	18 772 (2.3)	776 (2.3)	18 861 (2.3)	687 (2.6)	18 419 (2.3)	1129 (1.8)
Race and ethnicity, No. (%)							
American Indian and Native Alaskan	3004 (0.4)	2980 (0.4)	24 (0.1)	2989 (0.4)	15 (0.1)	2869 (0.4)	135 (0.2)
Asian	43 706 (5.1)	32 735 (4.0)	10 971 (32.3)	34 819 (4.2)	8887 (34.3)	25 185 (3.2)	18 521 (29.1)
Black and African American	76 671 (9.0)	67 276 (8.2)	9395 (27.7)	70 002 (8.5)	6669 (25.7)	55 883 (7.1)	20 788 (32.7)
Hispanic and Latino	34 562 (4.1)	25 388 (3.1)	9174 (27.0)	27 293 (3.3)	7269 (28.0)	24 928 (3.2)	9634 (15.1)
Native Hawaiian and Pacific Islander	840 (0.1)	807 (0.1)	33 (0.1)	814 (0.1)	26 (0.1)	687 (0.1)	153 (0.2)
White	659 671 (77.5)	657 363 (80.4)	2308 (6.8)	658 142 (79.7)	1529 (5.9)	649 884 (82.5)	9787 (15.4)
Multiracial	9984 (1.2)	9270 (1.1)	714 (2.1)	9443 (1.1)	541 (2.1)	8094 (1.0)	1890 (3.0)
Other^a^	12 732 (1.5)	11 653 (1.4)	1079 (3.2)	11 946 (1.4)	786 (3.0)	10 071 (1.3)	2661 (4.2)
Missing	10 240 (1.2)	9968 (1.2)	272 (0.8)	10 035 (1.2)	205 (0.8)	10 184 (1.3)	56 (0.1)

Abbreviation: NA, not applicable.

^a^ Race and ethnicity and preferred language were self-identified. Patient race options were prepopulated including the following categories: American Indian and Alaskan Native, Asian, Black and African American, Hispanic and Latino, Native Hawaiian and Pacific Islander, White, and other. Ethnicity options were dichotomous between Hispanic and Latino or not Hispanic and Latino. Patients could select more than 1 race; those individuals were categorized into 1 cohort as multiracial. Other was a prepopulated option for the registration questions on race and preferred language.

**Figure 1.**
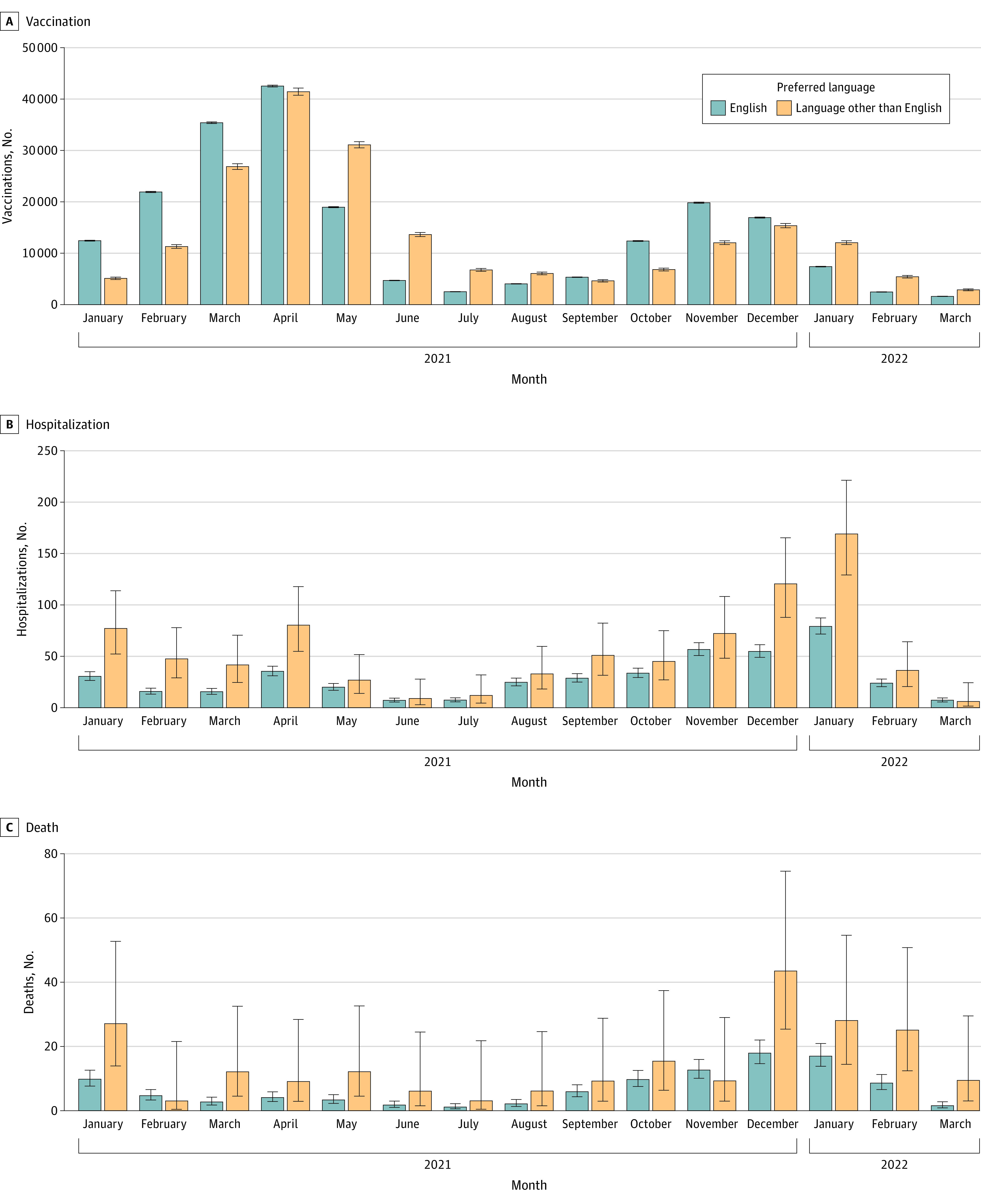
Age-Standardized Rates of Vaccination, Hospitalization, and Death in Individuals With vs Without Language Preference Other Than English Age-standardized monthly rates of vaccination (A), hospitalization (B), and death (C) by language preference per 100 000 individuals between January 2021 and March 2022. Rates are standardized to the age distribution of the overall patient population. Point estimates and 95% CIs were derived from Poisson regression models adjusted by age group and the interaction of month and language preference. Error bars indicate 95% CIs.

**Figure 2.**
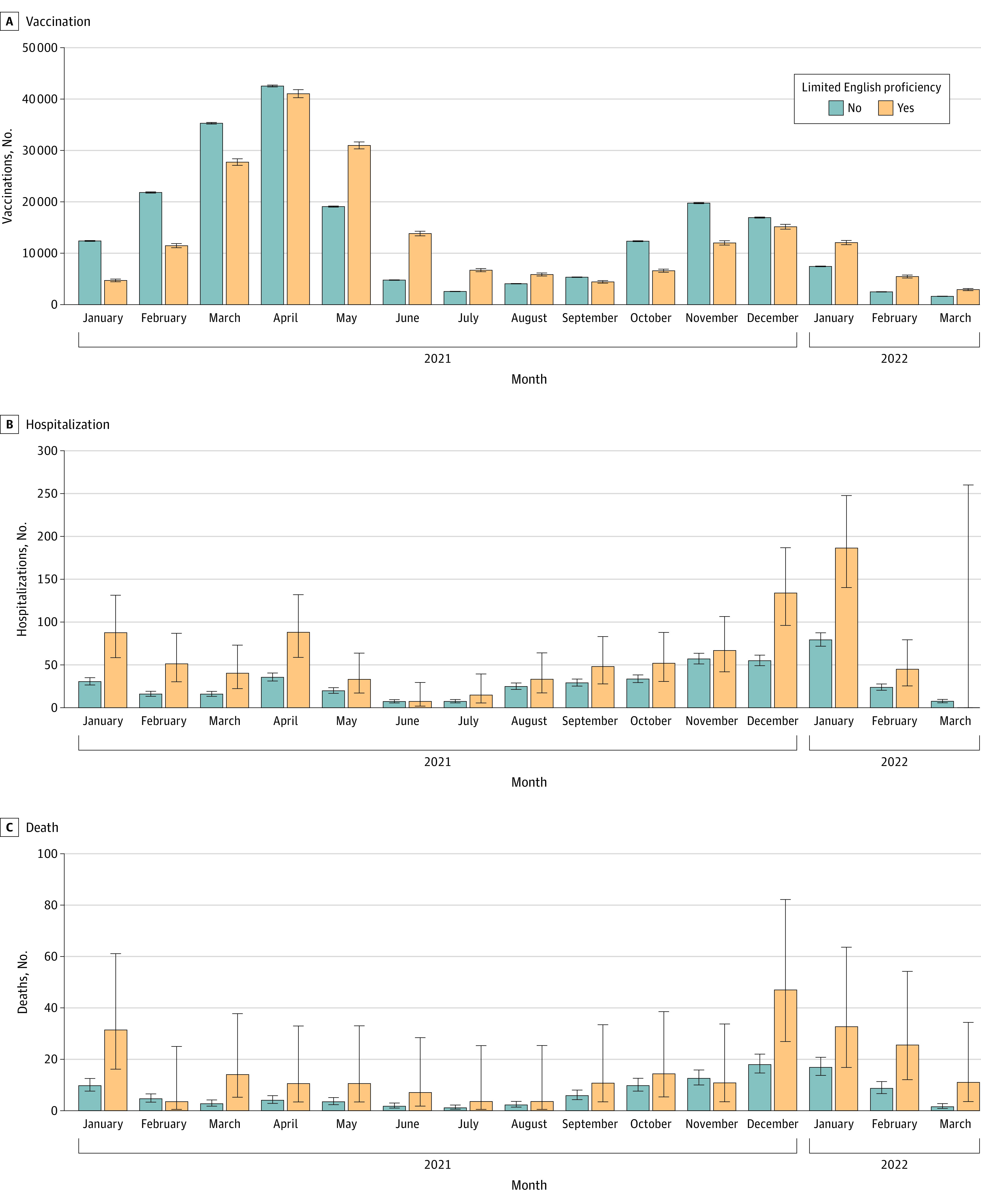
Age-Standardized Rates of Vaccination, Hospitalization, and Death in Individuals With vs Without Limited English Proficiency Age-standardized monthly rates of vaccination (A), hospitalization (B), and death (C) by English proficiency as measured by interpreter need per 100 000 patients between January 2021 and March 2022. Rates are standardized to the age distribution of the overall study population. Point estimates and 95% CIs were derived from Poisson regression models adjusted by age group and the interaction of month and English proficiency. Error bars indicate 95% CIs.

Marked temporal clusters were observed for COVID-19 vaccination uptake, hospitalization, and death, represented by the monthly age-adjusted rates per 100 000 individuals for LPOE (Figure 1) and LEP (Figure 2). January through May 2021 corresponded to the rollout of the COVID-19 vaccine primary series and the Alpha and Delta COVID-19 variants; October 2021 through March 2022 coincided with the booster vaccine and the Omicron variant. A delay in vaccination of both primary series and booster uptake was observed for the LPOE and LEP cohorts (Figure 1 and Figure 2). In comparison, hospitalizations and deaths occurred more frequently across those groups throughout the study period (Figure 1 and Figure 2).

By the end of the observation period, vaccine rates did not vary greatly for most groups by LPOE and LEP compared with the reference group (RR, 0.96; 95%; 95% CI, 0.95-0.96). However, delayed vaccine uptake was observed with LPOE (HR, 0.83; 95% CI, 0.82-0.84) and LEP (HR, 0.81; 95% CI, 0.80-0.82). This delay was inversely associated with increased COVID-19 hospitalization and death rates. Patients with LPOE had approximately twice the risk of hospitalization (RR, 1.85; 95% CI, 1.63-2.08) and death (RR, 2.13; 95% CI, 1.65-2.69) (Table 2). Patients with LEP had an even higher risk of hospitalization (RR, 1.98; CI, 1.73-2.25) and COVID-19–associated death (RR, 2.32; 95% CI, 1.79-2.95) compared with those not needing interpreters (Table 2). We reanalyzed the associations after undersampling the reference group and found the reported HRs and RRs were stable across imbalances in population sizes.

**Table 2.** Subgroup-Specific Primary Outcome of COVID-19 Vaccination Uptake With Time to First Vaccine Dose and Vaccination Rates and Secondary Outcomes

Group	No.	Time to first vaccine, HR (95% CI)	Vaccination, age-adjusted, RR (95% CI)	Hospitalization, age-adjusted, RR (95% CI)	Death, age-adjusted, RR (95% CI)
Language preference other than English	33 970	0.83 (0.82-0.84)	0.96 (0.95-0.96)	1.85 (1.63-2.08)	2.13 (1.65-2.69)
Interpreter need	25 927	0.81 (0.80-0.82)	0.96 (0.95-0.96)	1.98 (1.73-2.25)	2.32 (1.79-2.95)
Birthplace other than US	63 625	1.02 (1.01-1.03)	1.03 (1.03-1.04)	1.70 (1.53-1.87)	1.65 (1.31-2.05)
Preferred language					
Arabic	593	0.71 (0.65-0.78)	0.87 (0.82-0.92)	0.39 (0.02-1.73)	3.80 (0.63-11.74)
Cambodian/Khmer	1266	1.08 (1.02-1.14)	1.17 (1.13-1.21)	2.22 (1.28-3.54)	2.74 (0.98-5.91)
Chinese languages^b^	1329	1.06 (1-1.12)	1.14 (1.10-1.17)	0.83 (0.33-1.67)	1.44 (0.36-3.74)
Eastern European languages	1146	0.43 (0.40-0.46)	0.68 (0.65-0.71)	2.76 (1.72-4.17)	3.51 (1.68-6.37)
Ethiopian languages	2460	0.84 (0.81-0.88)	0.91 (0.89-0.94)	1.48 (0.85-2.36)	NA
Hmong	1746	0.89 (0.84-0.93)	1.00 (0.97-1.04)	3.05 (2.02-4.39)	5.48 (2.92-9.24)
Myanmar (formerly Burma) languages	909	0.73 (0.68-0.79)	0.96 (0.91-1.00)	2.70 (1.29-4.87)	3.62 (0.60-11.22)
South Asian languages	579	1.24 (1.14-1.35)	1.16 (1.10-1.22)	NA	NA
Laotian	618	0.97 (0.90-1.06)	1.10 (1.05-1.16)	1.73 (0.69-3.50)	5.40 (1.93-11.65)
Nepali	584	1.09 (1.00-1.18)	1.21 (1.15-1.27)	3.03 (1.30-5.86)	NA
American Sign Language	712	0.99 (0.91-1.07)	1.03 (0.98-1.08)	2.66 (1.28-4.81)	5.06 (1.57-11.79)
Somali	6562	0.58 (0.57-0.60)	0.72 (0.71-0.74)	2.12 (1.60-2.75)	1.31 (0.52-2.67)
Spanish	9166	0.85 (0.83-0.87)	0.97 (0.96-0.99)	1.99 (1.55-2.5)	1.87 (0.97-3.23)
Vietnamese	3511	1.14 (1.10-1.18)	1.13 (1.11-1.16)	1.16 (0.73-1.74)	2.19 (1.09-3.85)
Other (n <500)	2789	0.99 (0.95-1.03)	1.01 (0.99-1.04)	1.36 (0.83-2.06)	0.52 (0.09-1.61)
Race and ethnicity					
American Indian and Native Alaskan	3004	0.92 (0.88-0.96)	0.92 (0.90-0.94)	2.19 (1.41-3.21)	4.01 (1.83-7.51)
Asian	43 706	1.22 (1.21-1.23)	1.16 (1.15-1.17)	1.23 (1.05-1.42)	1.98 (1.48-2.6)
Black and African American	76 671	0.71 (0.70-0.71)	0.82 (0.81-0.82)	2.65 (2.43-2.90)	1.80 (1.39-2.3)
Hispanic and Latino	34 562	0.88 (0.87-0.89)	0.96 (0.95-0.97)	2.08 (1.80-2.39)	1.82 (1.22-2.62)
Native Hawaiian and Pacific Islander	840	0.94 (0.87-1.01)	0.97 (0.92-1.01)	1.99 (0.79-4.03)	1.55 (0.09-6.83)
Multiracial	9984	0.97 (0.95-1.00)	0.99 (0.97-1.00)	1.83 (1.38-2.36)	2.76 (1.55-4.50)
Other^a^	12 732	0.90 (0.88-0.92)	0.96 (0.95-0.97)	0.91 (0.66-1.23)	0.79 (0.34-1.54)
Missing	10 240	0.75 (0.73-0.77)	0.84 (0.83-0.85)	0.09 (0.02-0.24)	2.19 (1.23-3.57)

Abbreviations: HR, hazard ratio; RR, rate ratio.

^a^ Race, ethnicity and preferred language were self-identified. Patient race options were prepopulated including the following categories: American Indian and Alaskan Native, Asian, Black and African American, Hispanic and Latino, Native Hawaiian and Pacific Islander, White, and other. Ethnicity options were dichotomous between Hispanic and Latino or not Hispanic and Latino. Patients could select more than 1 race; those individuals were categorized into 1 cohort as multiracial. Other was a prepopulated option for the registration questions on race and preferred language.

^b^ Inclusive of Mandarin, Cantonese, Chinese, and Taishanese.

Comparative analysis among traditional race and ethnicity groups with White race as the reference group revealed delayed vaccine uptake for Black (HR 0.71; 95% CI, 0.70-0.71), Hispanic (HR, 0.88; 95% CI, 0.87-0.89), Native American (HR, 0.92; 95% CI, 0.88-0.96), Native Hawaiian/Pacific Islander (HR, 0.94; 95% CI, 0.87-1.01), other (HR, 0.90; 95% CI, 0.88-0.92), and multiracial (HR, 0.97; 95% CI, 0.95-1.00). Conversely, the Asian cohort had faster vaccine uptake (HR, 1.22; 95% CI, 1.21-1.23). Vaccination rate was lowest for the Black (RR, 0.82; 95% CI, 0.81-0.82) and highest for Asian (RR, 1.16; 95% CI, 1.15-1.17) cohorts. Hospitalizations were higher in most cohorts compared with the White group, notably, 2.6 times higher for the Black group (RR, 2.65; 95% CI, 2.43-2.90); 2.1 times higher for the Hispanic group (RR, 2.08; 95% CI, 1.80-2.39); 2.2 times higher for the Native American group (RR, 2.19; 95% CI, 1.41-3.21), and 2 times higher for Native Hawaiian/Pacific Islander group (RR, 1.99; 95% CI, 0.79-4.03). Deaths followed a similar pattern, with COVID-19–associated mortality 2 times higher for the Asian (RR, 1.98; 95% CI, 1.48-2.60), Black (RR, 1.8; 95% CI, 1.39-2.30), and Hispanic (RR, 1.82; 95% CI, 1.22-2.62) cohorts; almost 3 times higher for the multiracial cohort (RR, 2.76; 95% CI, 1.55-4.50); and 4 times higher for Native American cohort (RR, 4.01; 95% CI, 1.83-7.51) (Table 2).

**Figure 3.**
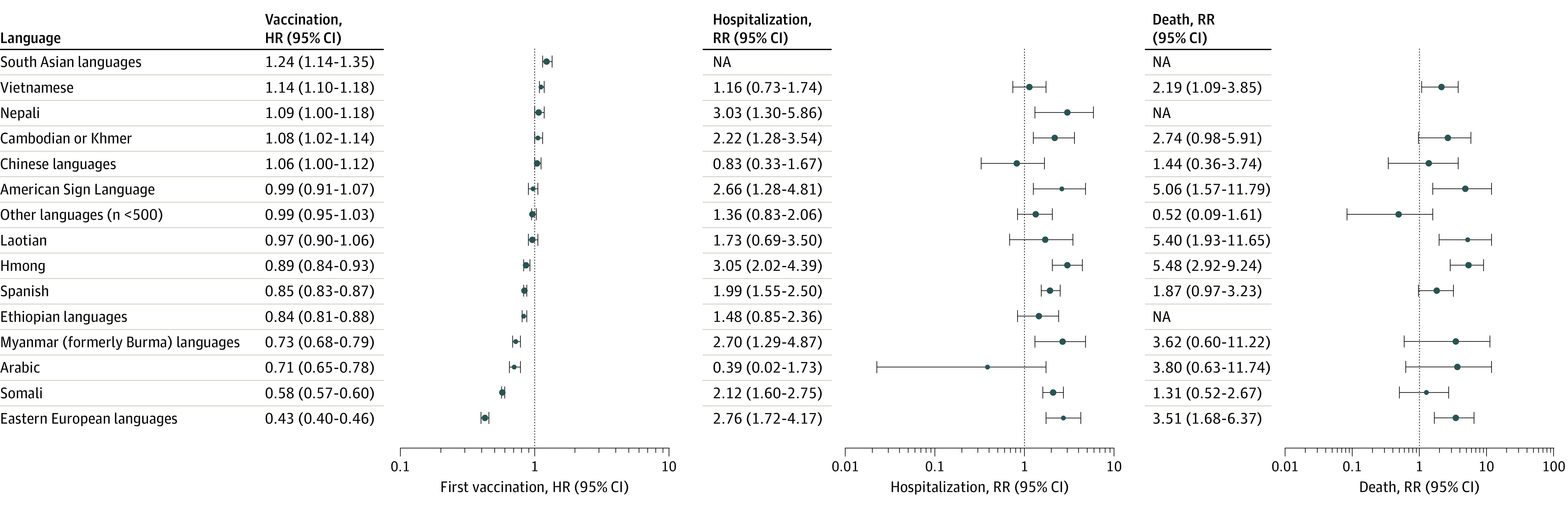
Age-Standardized Rates for Time to First Vaccine, Hospitalization, and Death in Individuals by Language Preference Groupings Hazard ratios (HRs) of time to first vaccine (A) and rate ratios (RRs) of hospitalization (B) and death (C) by language preference groupings. Error bars indicate 95% CIs. NA indicates not applicable.

Several language groups had delayed vaccinations compared with English-speaking counterparts. Variation was noteworthy in outcomes for language groups that would traditionally have been aggregated under race and ethnicity categorization. For example, Hmong (Asian category) had delayed vaccine uptake (HR, 0.89; 95% CI, 0.84-0.93), but by the end of the observation period had similar overall vaccination rates (RR, 1.00; 95% CI, 0.97-1.04). This vaccination pattern was associated with a higher rate of hospitalization (RR, 3.05; 95% CI, 2.02-4.39) and death (RR, 5.48; 95% CI, 2.92-9.24). Similarly, Myanmar (formerly Burma) languages had delayed vaccine uptake (HR, 0.73; 95% CI, 0.68-0.79) but achieved similar overall vaccination rates by the end of the study period (RR, 0.96; 95% CI, 0.91-1.00). This delay was again associated with higher hospitalization (RR, 2.70; 95% CI, 1.29-4.87) and death (RR, 3.62; 95% CI, 0.60-11.22) rates. Conversely, faster vaccine uptake in certain language groups was not associated with fewer hospitalizations and deaths (Figure 3).

A pronounced delay in vaccination uptake was observed in Eastern European languages (White category) (HR, 0.43; 95% CI, 0.40-0.46), with a decreased overall vaccination rate (RR, 0.68; 95% CI, 0.65-0.71) and increased COVID-19 hospitalization (RR, 2.76; 95% CI, 1.72-4.17) and death (RR, 3.51; 95% CI, 1.68-6.37) rates. Somali speakers (Black category) had delayed vaccination (HR, 0.58; 95% CI, 0.57-0.60) uptake, decreased overall vaccination rate (RR, 0.72; 95% CI, 0.71-0.74), and increased hospitalization (RR, 2.12; 95% CI, 1.60-2.75) and death (RR, 1.31; 95% CI, 0.52-2.67) rates. Spanish speakers also had delayed vaccine uptake (HR, 0.85; 95% CI, 0.83-0.87), decreased vaccination rates (RR, 0.97; 95% CI, 0.96-0.99) and increased COVID-19–associated hospitalization (RR, 1.99; 95% CI, 1.55-2.50) and death (RR, 1.87; 95% CI, 0.97-3.23) rates.

## Discussion

Although by the end of the study period most LPOE and LEP groups had similar overall vaccine uptake rates, temporal clusters in the study population, marked by delayed time to first COVID-19 vaccine dose, were associated with increased COVID-19–associated hospitalizations and deaths. COVID-19 clinical outcomes associated with LPOE and LEP would have remained unexplained without this temporal perspective. Delayed time to vaccine and worse health outcomes patterns suggest LPOE and LEP as a proxy for limited language access are crucial risk factors influencing health disparities.

Although racial and ethnic categories remain the most common method for describing US health disparities, they provide minimal direct intervention information. Systematically collecting data on LPOE and LEP could enhance understanding of observed disparities while providing actionable information to address inequities. Grouping by language preference offers one method to identify the need for linguistically aligned health care resources for effective communication. It can augment local knowledge to identify higher-risk social groups for engaging trusted messengers, forging community partnerships, developing culturally appropriate interventions, and delivering messages in an acceptable, linguistically congruent medium. This lack of LPOE and LEP data limits public health and health care systems in addressing disparities, particularly during a public health emergency, when delays lead to increased morbidity and mortality.

Prior studies have considered language groups to identify themes of vaccine hesitancy and health preferences.^16,22^ A systematic review focused on racial and ethnic minority groups and migrants found barriers associated with language and reduced physical access to COVID-19 vaccines.^17^ Linguistic diversity—particularly in regions with smaller representation of LPOE—exacerbates language access barriers.^18^ Prior studies have named systemic, institutional, and structural racism as systemic factors for disparities in vaccine uptake in minoritized groups.^3,4,5,6,7,10,18,23^ Many qualitative studies have highlighted the value of engaging individuals within cultural communities for collective knowledge of best practice techniques for communication and outreach.^14,15,16,17,19,22,23,24^

Patient, community or social group, and health-system–associated factors may influence vaccine uptake or hesitancy. Language preference correlates with cultural health beliefs, social determinants of health and barriers to care in refugee, immigrant, and migrant communities.^13^ The Minnesota Department of Health created neighborhood-based vaccination sites, supported community-based organizations’ staff to become COVID-19 community coordinators, and addressed privacy, multilingual access, and disability access.^25,26^ More detailed LPOE and LEP data could have increased engagement with groups experiencing disparities. The National Resource Center for Refugees, Immigrants, and Migrants and community-based organizations created multimedia communications while engaging faith leaders and youth ambassadors.^27^ Local and national vaccine campaigns may have affected regional vaccine uptake. Linguistically aligned vaccine campaigns may have improved uptake in the study population compared with other areas in the US. Improved preparedness by health systems and local health departments should include linguistically and culturally concordant materials and dissemination with community-based organization partners. Data allowing focused efforts on social groups with the greatest risk early in a pandemic response may improve health outcomes and reduce disparities.

### Limitations

Our study has many important limitations. We were unable to access Wisconsin death records; thus, deaths that occurred in Wisconsin were not represented. The results are not necessarily generalizable to the US population, as this health care system comprises a unique combination of cultures and languages. The vaccine eligibility date for the cohort was set as December 15, 2020. Based on age distribution, professional risk categories, and comorbidities, certain populations were eligible for the vaccine earlier than others, which may have skewed results (this was not adjusted for in the analysis). COVID-19 hospitalizations outside of the health system could be undercounted. Vaccination data only represented the mean vaccines received per person in each social group but did not capture whether patients completed the initial vaccine series or received a booster vaccine. However, most patients who received messenger RNA vaccines completed the primary series; as such, the first dose was a reasonable surrogate for completion of the primary series. In the absence of comprehensive disaggregated ethnicity options for this health system, a subgroup of patients who may associate with a cultural group but have high English language proficiency and English language preference was not represented; however, this likely would have made our outcomes more conservative. Notably, there were no COVID-19–associated deaths in some language groups, including Nepali, South Asian, and Ethiopian languages. This may have been related to small representation of these groups in the data set. Missing data on main exposures, including country of birth, language preference, and interpreter need, may have introduced bias, as nonresponders were placed in dominant categories for each variable.

## Conclusions

In this study, delayed time to first COVID-19 vaccine and subsequent increased COVID-19 hospitalizations and mortality were seen in LPOE and LEP groups compared with the English-language reference group. Traditional race and ethnicity analysis of health disparities may obscure social groups at higher risk. The findings presented herein suggest a language justice framework to collect data on language preference and interpreter need offers opportunities to redistribute resources such as translated materials and medical interpreter services, address barriers to care for disproportionately affected LPOE and LEP groups, and provide actionable information to address health disparities during a public health emergency.

## Notes

Supplement 1. **eTable.** Additional Baseline Characteristics of Language Preference Other Than English (LPOE), Sex, Age by Race/Ethnicity **eMethods.** Notes on Methodology
eReferences

Supplement 2.
Data Sharing Statement
