# Supplementary material for: Evaluation of Preferred Language and Timing of COVID-19 Vaccine Uptake and Disease Outcomes
Source: JAMA Netw Open. 2023 Apr 12;6(4):e237877. doi: 10.1001/jamanetworkopen.2023.7877 (PMC10099068; doi:10.1001/jamanetworkopen.2023.7877)
Supplement: Supplement 1. — eTable. Additional Baseline Characteristics of Language Preference Other Than English (LPOE), Sex, Age by Race/Ethnicity eMethods. Notes on Methodology eReferences [file jamanetwopen-e237877-s001.pdf]

Supplementary Online Content

Quadri NS, Knowlton G, Vazquez Benitez G, et al. Evaluation of preferred language and timing of COVID-19 vaccine uptake and disease outcomes. *JAMA Netw Open*. 2023;6(4):e237877. doi:10.1001/jamanetworkopen.2023.7877

eTable. Additional Baseline Characteristics of Language Preference Other Than English (LPOE), Sex, Age by Race/Ethnicity

eMethods. Notes on Methodology

eReferences

This supplementary material has been provided by the authors to give readers additional information about their work.

© 2023 Quadri NS et al. *JAMA Network Open.*

eTable. Additional Baseline Characteristics of Language Preference Other Than English (LPOE), Sex, Age by Race/Ethnicity

	White	Asian	Black/African American	Hispanic	Missing	Multiracial	American Indian/Native Alaskan	Other[1]	Native Hawaiian/Pacific Islander
N	659,671	43,706	76,671	34,562	10,240	9,984	3,004	12,732	840
Foreign-born (%)	9,787 (1.5)	18,521 (42.4)	20,788 (27.1)	9,634 (27.9)	56 (0.5)	1,890 (18.9)	135 (4.5)	2,661 (20.9)	153 (18.2)
Language Preference other than English (%)	2,306 (0.3)	10,971 (25.1)	9,395 (12.3)	9,174 (26.5)	272 (2.7)	714 (7.2)	24 (0.8)	1,079 (8.5)	33 (3.9)
Interpreter Need (%)	1,638 (0.2)	8,907 (20.4)	6,728 (8.8)	7,308 (21.1)	209 (2.0)	546 (5.5)	18 (0.6)	794 (6.2)	26 (3.1)
Preferred Language (%)									
English	657,365 (99.7)	32,735 (74.9)	67,276 (87.7)	25,388 (73.5)	9,968 (97.3)	9,270 (92.8)	2,980 (99.2)	11,653 (91.5)	807 (96.1)
Arabic	165 (0.0)	37 (0.1)	130 (0.2)	5 (0.0)	11 (0.1)	78 (0.8)	1 (0.0)	165 (1.3)	1 (0.1)
Cambodian/Khmer	2 (0.0)	1,195 (2.7)	1 (0.0)	10 (0.0)	1 (0.0)	21 (0.2)	0 (0.0)	33 (0.3)	3 (0.4)
Chinese Languages	6 (0.0)	1,276 (2.9)	1 (0.0)	4 (0.0)	4 (0.0)	19 (0.2)	0 (0.0)	17 (0.1)	2 (0.2)
Eastern European Languages	1,021 (0.2)	10 (0.0)	1 (0.0)	9 (0.0)	5 (0.0)	23 (0.2)	3 (0.1)	74 (0.6)	0 (0.0)
Ethiopian Languages	4 (0.0)	5 (0.0)	2,198 (2.9)	12 (0.0)	22 (0.2)	127 (1.3)	1 (0.0)	90 (0.7)	0 (0.0)
Hmong	4 (0.0)	1,659 (3.8)	0 (0.0)	4 (0.0)	27 (0.3)	16 (0.2)	2 (0.1)	34 (0.3)	0 (0.0)
Burma/Myanmar Languages	2 (0.0)	873 (2.0)	2 (0.0)	1 (0.0)	15 (0.1)	10 (0.1)	0 (0.0)	5 (0.0)	1 (0.1)
South Asian Languages	9 (0.0)	409 (0.9)	9 (0.0)	2 (0.0)	6 (0.1)	50 (0.5)	8 (0.3)	81 (0.6)	5 (0.6)
Laotian	2 (0.0)	591 (1.4)	1 (0.0)	4 (0.0)	4 (0.0)	7 (0.1)	0 (0.0)	8 (0.1)	1 (0.1)
Nepali	8 (0.0)	502 (1.1)	3 (0.0)	6 (0.0)	3 (0.0)	41 (0.4)	0 (0.0)	20 (0.2)	1 (0.1)
Sign language	462 (0.1)	929 (2.1)	1,010 (1.3)	100 (0.3)	34 (0.3)	119 (1.2)	0 (0.0)	122 (1.0)	12 (1.4)
Somali	538 (0.1)	49 (0.1)	75 (0.1)	24 (0.1)	7 (0.1)	3 (0.0)	3 (0.1)	11 (0.1)	2 (0.2)
Spanish	26 (0.0)	33 (0.1)	5,955 (7.8)	11 (0.0)	34 (0.3)	162 (1.6)	5 (0.2)	334 (2.6)	2 (0.2)
Vietnamese	38 (0.0)	2 (0.0)	9 (0.0)	8,966 (25.9)	88 (0.9)	4 (0.0)	1 (0.0)	58 (0.5)	0 (0.0)

© 2023 Quadri NS et al. *JAMA Network Open.*

Other Language (n < 500)	19 (0.0)	3,401 (7.8)	0 (0.0)	16 (0.0)	11 (0.1)	34 (0.3)	0 (0.0)	27 (0.2)	3 (0.4)
Female	380,438 (57.7)	26,395 (60.4)	44,813 (58.4)	20,720 (60.0)	5,940 (58.0)	6,066 (60.8)	2,057 (68.5)	6,996 (54.9)	485 (57.7)
Male	279,233 (42.3)	17, 311 (39.6)	31,858 (41.6)	13,842 (40.0)	4,300 (42.0)	3,918 (39.2)	947 (31.5)	5,736 (45.0)	355 (42.3)
Age (mean (SD))	51.65 (18.4)	44.4 (16.5)	42.4 (15.8)	41.1 (15.7)	42.8 (17.5)	40.9 (16.8)	46.9 (16.5)	45.2 (17.6)	46.8 (17.21)
Age Group (%)									
18-30	103,563 (15.7)	9,433 (21.6)	19,898 (26.0)	10,529 (30.5)	2,974 (29.0)	3,259 (32.6)	581 (19.3)	2,967 (23.3)	171 (20.4)
31-50	202,814 (30.7)	19,974 (45.7)	33,779 (44.1)	14,641 (42.4)	4,115 (40.2)	3,986 (39.9)	1,142 (38.0)	5,119 (40.2)	331 (39.4)
51-64	186,990 (28.3)	8,888 (20.3)	16,098 (21.0)	6,778 (19.6)	1,946 (19.0)	1,735 (17.4)	848 (28.2)	2,731 (21.4)	203 (24.2)
65-84	148,366 (22.5)	4,912 (11.2)	6,468 (8.4)	2,435 (7.0)	1,011 (9.9)	913 (9.1)	413 (13.7)	1,731 (13.6)	120 (14.3)

[1] Race, ethnicity and preferred language were self-identified. Patient race options were pre-populated including the following categories: Black/African American, Asian, American Indian/Alaskan Native, Hispanic/Latino, Native Hawaiian/Pacific Islander, White, and Other. Additionally, ethnicity options were dichotomous between Hispanic/Latino or not Hispanic/Latino. Patients could select more than one race; those patients were categorized into one cohort as Multiracial. Other was a pre-populated option for the registration questions on race and preferred language.

© 2023 Quadri NS et al. *JAMA Network Open.*

eMethods. Notes on Methodology

The National Resource Center for Refugees, Immigrants, and Migrants (NRC-RIM) was created to provide linguistically and culturally appropriate intervention materials, facilitate public and private partnerships, identify community trusted messengers, and to collect and disseminate best practices during the COVID-19 pandemic. HealthPartners was chosen as a partner in this work because the organization has routinely collected Country of Birth (COB) and preferred language information as part of patient registration since 2005.

According to the US Census bureau, linguists recognize over 7,000 world languages which require coding for feasible tabulation, with aggregation based on population size. Aggregation methods consider the utility of the grouping for research and election requirements for voting rights.[1] We considered this methodology with application to languages in the study population. Similarly, sample size supported determination of disaggregated stand-alone language preference categories and those that required aggregation. Attention was given to disaggregated groups representing established refugee populations, as well as grouping languages of a specific region or country. The goal of aggregation was inclusion of the majority of the languages represented in the dataset within the above framework.

Disaggregated languages listed from most common to least common in the dataset

*Denotes well-established refugee groups in the region[2]

English

Spanish

Somali*

Vietnamese*

Hmong*

Cambodian/Khmer*

Sign Language

Laotian

Arabic

Nepali*

Aggregated languages listed from most common to least common in the dataset and delineating largest to smallest subgroup within a language category from left to right

*Denotes well-established refugee groups in the region[2]

Ethiopian Languages: Oromo*, Amharic*, Anuak*, Tigrinya

Eastern Europe languages: Russian*, Bosnian, Ukrainian, Romanian, Polish, Bulgarian, Albanian, Serbian

Chinese Languages: Mandarin, Cantonese, Chinese, Taishanese

Myanmar (formerly Burma) Languages: Karen*, Burmese

South Asian Languages: Hindi, Telugu, Bengali, Tamil, Gujarati, Urdu, Punjabi, Malayalam

Other languages (n < 500 participants): French, Korean, Swahili, Tibetan, Farsi, Tagalog, Thai, Japanese, Portuguese, Yoruba, Mandigo, Turkish, Pashto, Creole, German, Filipino, Twi, Bassa, Kissii, Ewe, Pidgin English, Indonesian, Igbo, Kinyarwanda, Nuer, Kpelleh, Krahn, Hebrew, Lingala

© 2023 Quadri NS et al. *JAMA Network Open.*

eReferences

1. 1. United States Census Bureau. About language use in the US population. December 16, 2021. Accessed August 25, 2022. https://www.census.gov/topics/population/language-use/about.html

   2. Minnesota Department of Health (MDH). Refugee health statistics. April 15 2022. Accessed October 5, 2022. https://www.health.state.mn.us/communities/rih/stats/index.html#Na
