# Supplementary material for: Evaluation of Preferred Language and Timing of COVID-19 Vaccine Uptake and Disease Outcomes
Source: JAMA Netw Open. 2023 Apr 12;6(4):e237877. doi: 10.1001/jamanetworkopen.2023.7877 (PMC10099068; doi:10.1001/jamanetworkopen.2023.7877)
Supplement: Supplement 2. — Data Sharing Statement [file jamanetwopen-e237877-s002.pdf]

Data Sharing Statement

Quadri. Evaluation of Preferred Language and Timing of COVID-19 Vaccine Uptake and Disease Outcomes. *JAMA Netw Open*. Published April 12, 2023.
doi:10.1001/jamanetworkopen.2023.7877

Data
Data available: No

Additional Information
Explanation for why data not available: The database constitutes almost the entire HealthPartners patient data and would include patients with unique identifiable information. We would be willing to share aggregate and de-identified data upon request.
